# Lemierre’s Syndrome: A Case Series

**DOI:** 10.7759/cureus.18436

**Published:** 2021-10-02

**Authors:** Tariq M Jaber, Vikram Saini, Osakpolor Ogbebor, Tiffany Dumont, Marvin Balaan, Mark Lega, Tariq Cheema

**Affiliations:** 1 Infectious Disease & Critical Care, Allegheny Health Network, Pittsburgh, USA; 2 Internal Medicine/Infectious Disease & Critical Care, Allegheny Health Network, Pittsburgh, USA; 3 Pulmonary and Critical Care Medicine, Allegheny Health Network, Pittsburgh, USA; 4 Pulmonary and Critical Care Medicine, Allegheny General Hospital, Pittsburgh, USA; 5 Division of Pulmonary and Critical Care Medicine, Allegheny General Hospital, Pittsburgh, USA

**Keywords:** lemierre’s syndrome, fusobacterium necrophorum, oropharyngeal infection, bacteremia, sepsis

## Abstract

Lemierre’s syndrome (LS) is a potentially fatal complication of oropharyngeal infection, resulting in contiguous suppurative thrombosis of the internal jugular vein (IJV) and septic emboli. It is most commonly associated with *Fusobacterium necrophorum (F. necrophorum)*, though other pathogens have also been implicated in its pathogenesis. The incidence of LS had so significantly decreased that it was referred to as "the forgotten disease." However, cases of LS have shown a resurgence, which may be partly attributed to an overreliance on a negative group A beta-hemolytic streptococcal rapid antigen detection test (RADT), commonly referred to as "rapid strep test." Clinicians must maintain a very high index of suspicion for LS in patients with persistent sequelae from tonsillopharyngitis who have a negative RADT.

## Introduction

In 1936, Andre Lemierre published a case series of 20 patients with "postanginal anaerobic septicemia" [subsequently termed "Lemierre’s syndrome" (LS)]; 18 of these cases turned out to be fatal [1]. LS is described as a post-pharyngeal infection complicated by the metastatic spread that culminates in septic shock with multi-organ failure [1-16]. Diagnosis is confirmed when radiographic imaging demonstrates internal jugular vein (IJV) thrombus with the recovery of an odontogenic pathogen [1-16]. LS is classically associated with *Fusobacterium necrophorum (F. necrophorum),* but other pathogens are also thought to be causal in its development, such as *Fusobacterium nucleatum*, *Streptococcus* species (spp.), *Staphylococcus* spp., and *Klebsiella pneumoniae* [4]. Standard treatment for LS includes antibiotic therapy with a primary focus on beta-lactam agents in addition to metronidazole [4]. The use of anticoagulants to combat LS remains controversial [3]. Surgical intervention is considered in cases of abscess formation and/or the persistence of a thrombus despite medical therapy [1,3].

The incidence of LS had declined significantly with the regular use of penicillin(s) [2,3,4]. However, a resurgence of the disease has recently been reported, perhaps owing to overreliance on a negative group A beta-hemolytic streptococcal rapid antigen detection test (RADT), commonly known as "rapid strep test," lack of disease awareness, and change in antibiotics prescription patterns in favor of a more conservative approach [4,5]. Additional factors that contributed to this resurgence may include antimicrobial resistance of the base pathogens involved, as well as a decrease in the number of tonsillectomies performed [2,4]. Some have even questioned if the resurgence represents a truly increased incidence versus a change in publication trends [5]. In light of this knowledge, it is critical to maintain a high index of suspicion in patients with tonsillitis and/or pharyngitis in the setting of persistent febrile illness, neck pain, and/or sepsis. In this case series, we highlight a recent resurgence of LS and the importance of early recognition and treatment in response to persistent symptoms and a negative RADT.

## Case presentation

Case 1

An 18-year-old male with a history of recurrent pharyngitis presented with a sore throat, fever, and progressive shortness of breath for 10 days. The patient had been previously evaluated at an outpatient urgent care clinic where both monospot and streptococcal RADT had been negative. He had been given a prescription for prednisolone for presumed bronchitis, which he had taken for two days prior to admission. He denied any history of sick contacts, travel, use of intravenous drugs, or recent dental intervention.

Upon admission to our hospital, the patient was febrile, tachycardic, hypotensive, and in respiratory distress. Physical exam revealed generalized neck pain aggravated by movement, right-sided swollen neck, and lymphadenopathy. The cardiac examination did not reveal any murmurs but pulmonary auscultation revealed bilateral coarse crackles. Abdominal, musculoskeletal, and neurological examinations were unremarkable.

Laboratory findings revealed the following results: white blood cell count: 24 k/µL (4.40-11.30 k/µL) with bandemia; platelet count: 26 k/µL (145-445 k/µL); and serum creatinine: 2.65 mg/dL (0.50-0.90 mg/dL). The liver function test was within the normal range.

**Figure 1:**
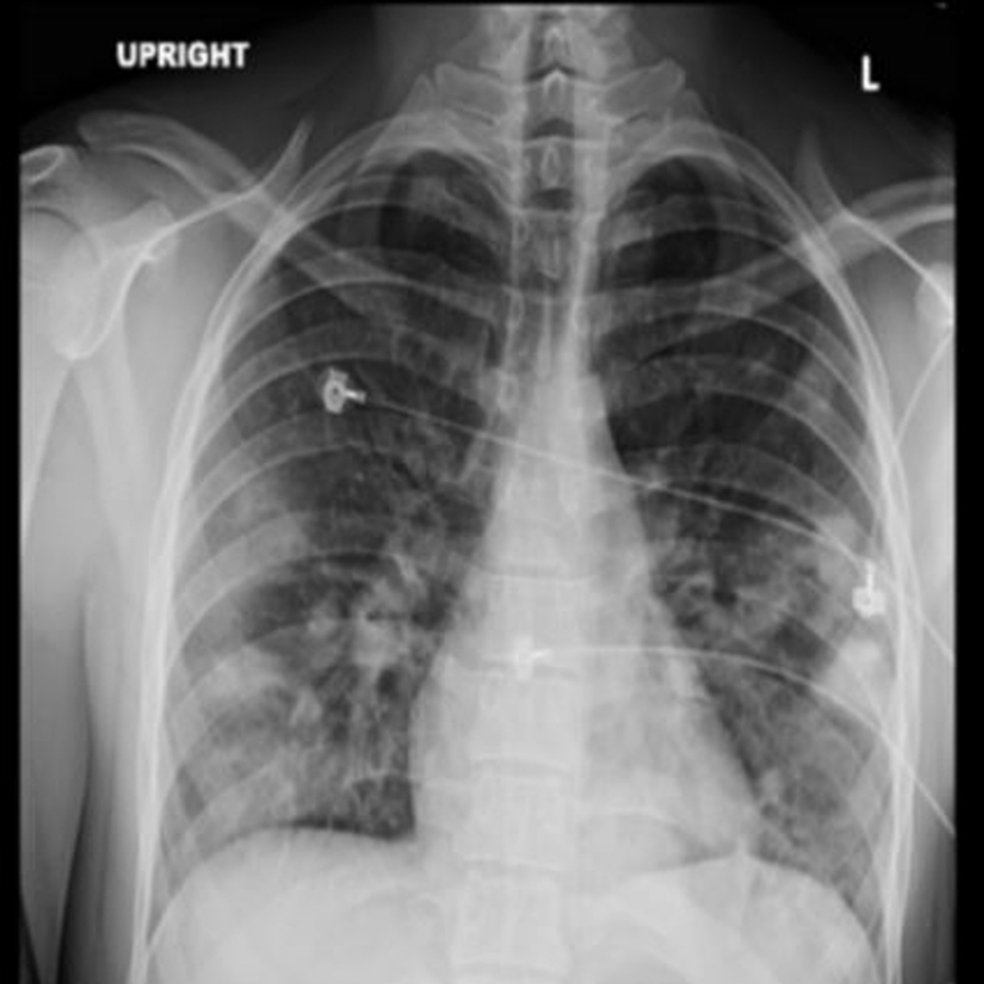
Chest X-ray Chest radiograph demonstrates multiple parenchymal opacities throughout both lungs in a peripheral distribution

**Figure 2:**
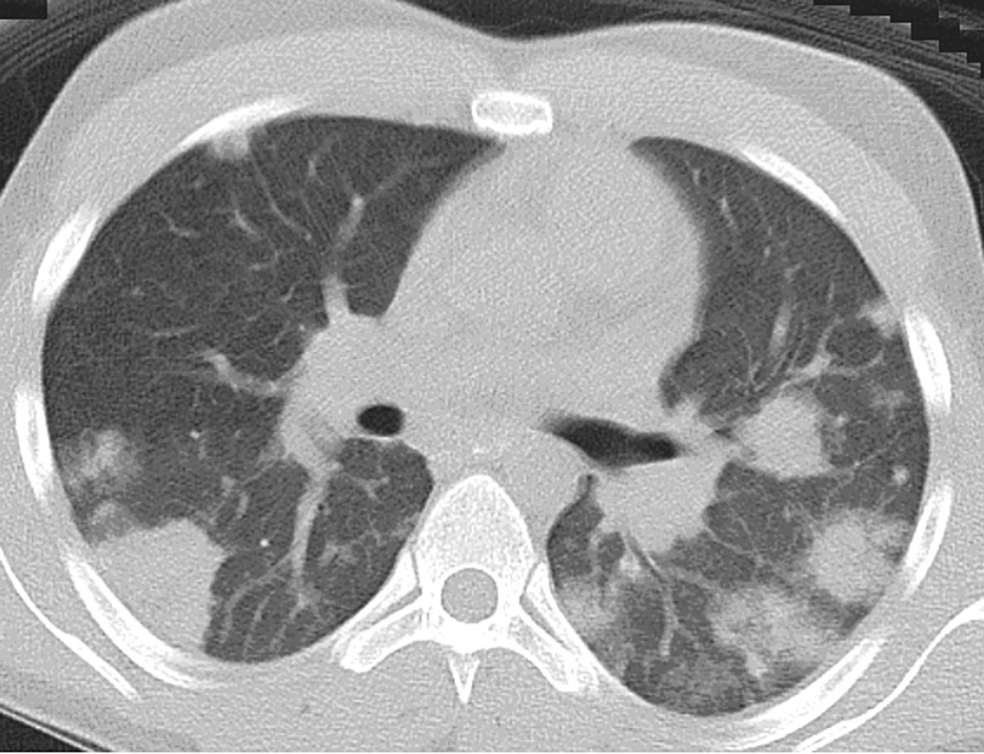
CT axial of the chest with IV contrast Select axial chest CT of the same patient delineates peripheral nodular and wedge-shaped opacities throughout both lungs corresponding to septic emboli CT: computed tomography; IV: intravenous

The patient’s condition deteriorated and he was emergently intubated. He developed acute respiratory distress syndrome (ARDS) and required prone positioning to allow for improved oxygenation. Chest radiograph (Figure 1) and subsequent CT scan (Figure 2) revealed multifocal pulmonary consolidation and nodules. A transesophageal echocardiogram was negative for valvular vegetations. Blood cultures revealed *F. necrophorum*, group F Streptococcus, and Gemella sp. Venous duplex ultrasound of the neck revealed a thrombus in the right IJV. The patient was subsequently diagnosed with LS. Intravenous ampicillin-sulbactam was initiated and a single dose of gentamicin was given. The patient subsequently defervesced, with extubation on day 18 of hospitalization, and was discharged on day 29 with an additional two-week course of amoxicillin-clavulanic acid. He did not receive anticoagulants. No further studies were performed for clot progression and/or resolution as the patient was lost to follow-up.

Case 2

A 20-year-old male with no past medical history presented to the urgent care center with a sore throat and lymphadenopathy for the past six days. The initial streptococcal RADT was negative. His symptoms did not improve and he was re-evaluated two days later at an outpatient clinic with fever and lethargy. At re-evaluation, a repeat streptococcal RADT was conducted, yielding negative results; the patient was subsequently sent home. The patient was found two days later (six days from initial onset) by a family member in a febrile state with altered mentation. Emergency services were called.

Upon arrival, the patient was hypotensive and in respiratory distress; he was rapidly intubated in the emergency department and required vasopressor support. Upon physical examination, he was noted to have a swollen neck. The cardiac exam did not reveal any murmurs, heaves, and/or gallops. However, the pulmonary exam demonstrated decreased air-on-entry, which was bilaterally worse on the right than the left. The abdominal examination did not reveal any nodularity and/or hepatosplenomegaly. The musculoskeletal exam was unremarkable.

**Figure 3:**
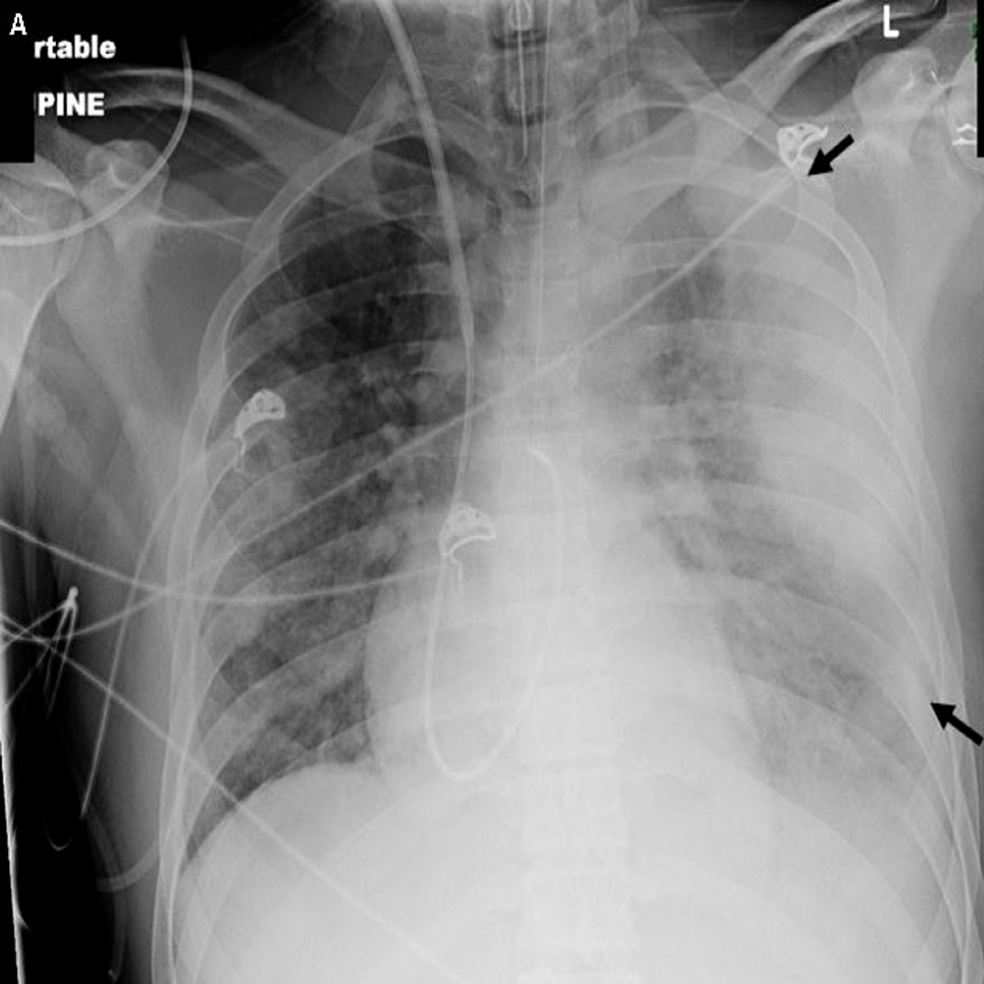
Chest X-ray AP chest radiograph demonstrates multiple nodular opacities throughout both lungs and a complex, partially loculated left pleural effusion (black arrows) AP: anteroposterior

**Figure 4:**
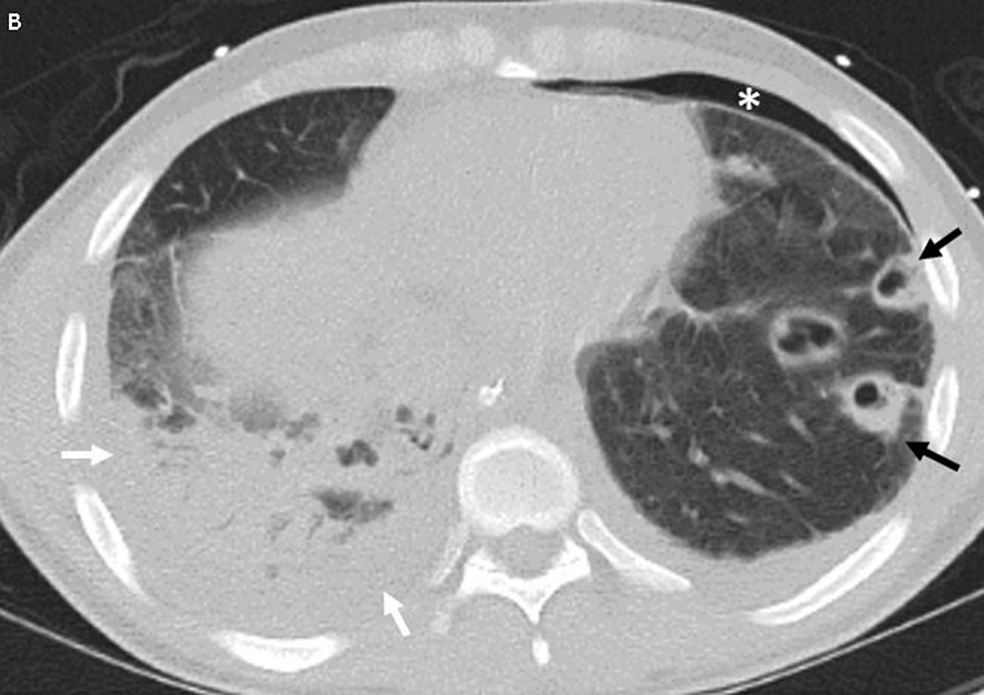
CT chest with IV contrast Select axial chest CT image of the same patient shows multiple cavitary septic embolic within the left lung (black arrows) along with a small pneumothorax (*) and small left pleural effusion. Airspace consolidation in the right lung (white arrows) corresponds to pneumonia with an adjacent small right pleural effusion CT: computed tomography; IV: intravenous

**Figure 5:**
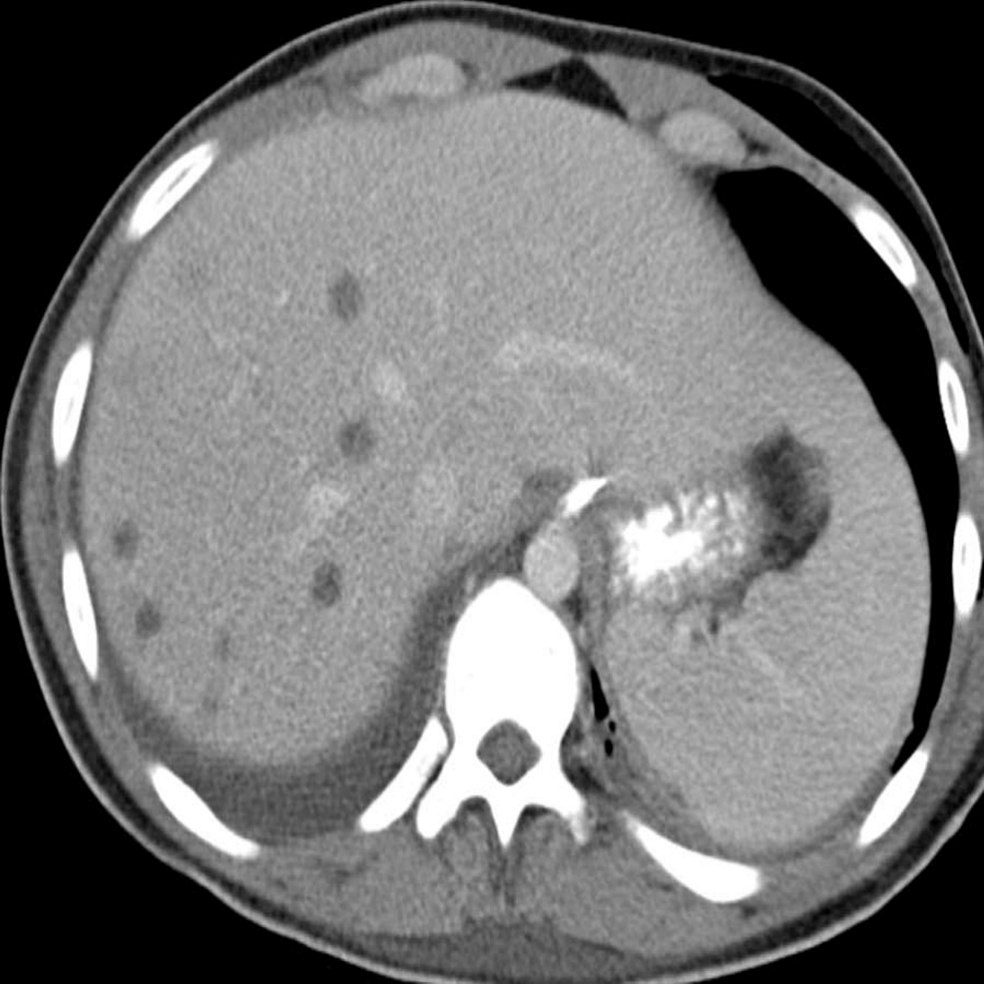
CT abdomen with IV and oral contrast Multiple hypodense lesions within the liver reflect developing septic emboli CT: computed tomography; IV: intravenous

**Figure 6:**
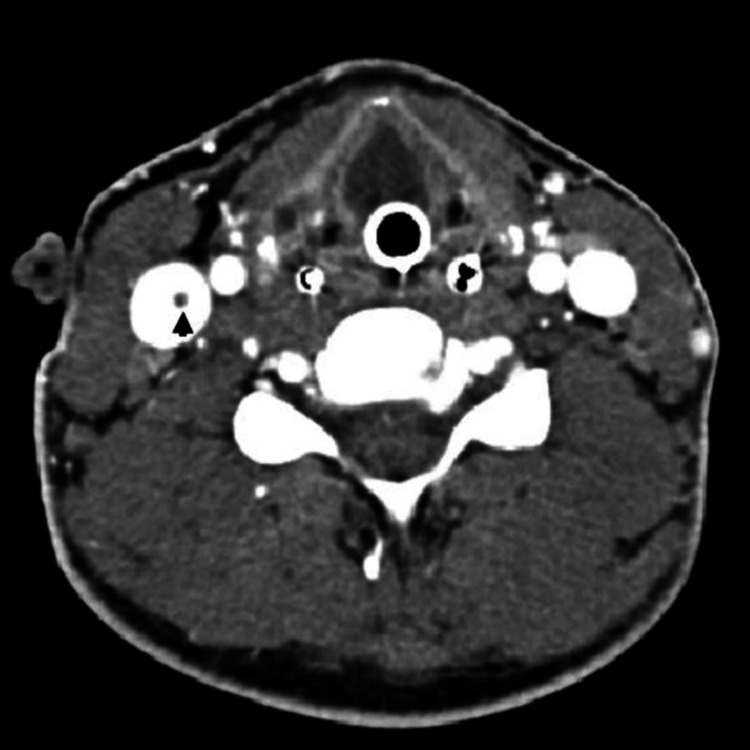
CT neck with IV contrast Axial contrast-enhanced CT of the neck shows a hypodense subocclusive thrombus within the right internal jugular vein (black arrow) CT: computed tomography; IV: intravenous

Laboratory data revealed the following results: white blood cell count: 19.35 k/µL (4.40-11.30 k/µL); platelets: 35 k/µL (145-445 k/µL); and serum creatinine: 2.07 mg/dL (0.50-0.90 mg/dL). Chest X-ray (Figure 3) and subsequent chest CT (Figure 4) showed a right pleural effusion and a loculated left pleural effusion with hydropneumothorax and nodular cavitary lesions. Abdominopelvic CT (Figure 5) demonstrated hepatic lesions representing small abscesses. Enhanced neck CT (Figure 6) revealed a small thrombus in the right IJV. Meropenem and vancomycin were initiated. A transesophageal echocardiogram was negative for cardiac vegetations. Blood cultures and pleural fluid cultures grew *F. necrophorum. *Meropenem and vancomycin were discontinued, and ampicillin-sulbactam was started. The patient’s hospital course was complicated by left bronchopleural fistula formation requiring multiple thoracotomy tube placements. He subsequently defervesced, was extubated on day 18, and discharged on day 32 of hospitalization (with an additional 11 days of amoxicillin-clavulanic acid). Anticoagulation therapy was not prescribed. The patient was lost to follow-up, and hence no data was available regarding clot progression and/or resolution.

Case 3

A 25-year-old previously healthy male presented to his primary care physician complaining of a two-day history of fevers, chills, sore throat, myalgias, and lymphadenopathy. Monospot and streptococcal RADT were negative. Antibiotics and corticosteroids were not prescribed. The patient’s symptoms progressed to nausea, emesis, cough, and weakness. He was admitted to a local hospital (outside facility) one week after the initial presentation with shortness of breath, productive cough, left-sided swollen neck with tenderness as well as signs of dehydration. Ceftriaxone and azithromycin were initiated at the community hospital for presumed community-acquired pneumonia.

**Figure 7:**
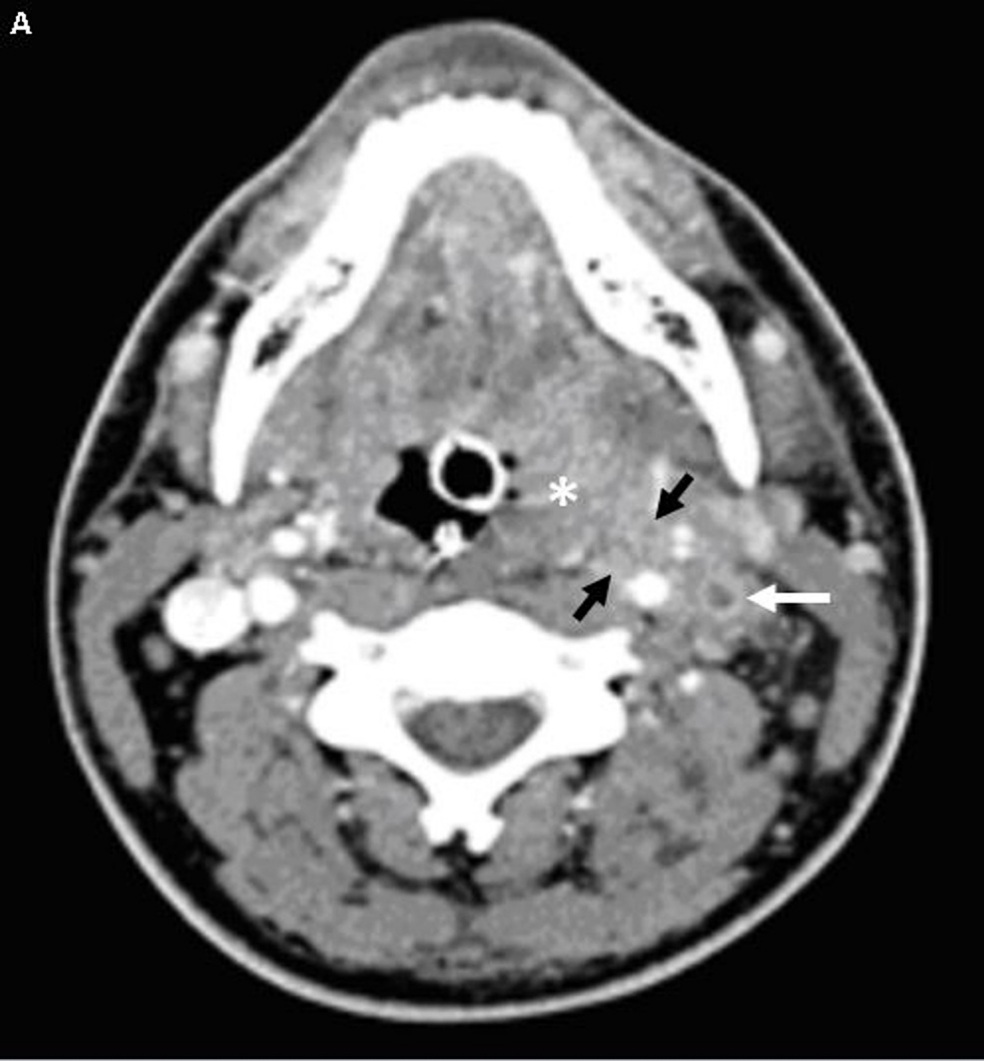
CT neck with IV contrast Axial contrast-enhanced CT of the neck shows an enlarged, hyperenhancing left palatine tonsil suggestive of acute tonsillitis (*). Inflammatory changes (black arrows) spread to the adjacent left carotid space with loss of the normal fat planes. Nonopacificiation of the left internal jugular with a hyperenhancing wall and surrounding soft tissue correspond to thrombosis and thrombophlebitis CT: computed tomography; IV: intravenous

**Figure 8:**
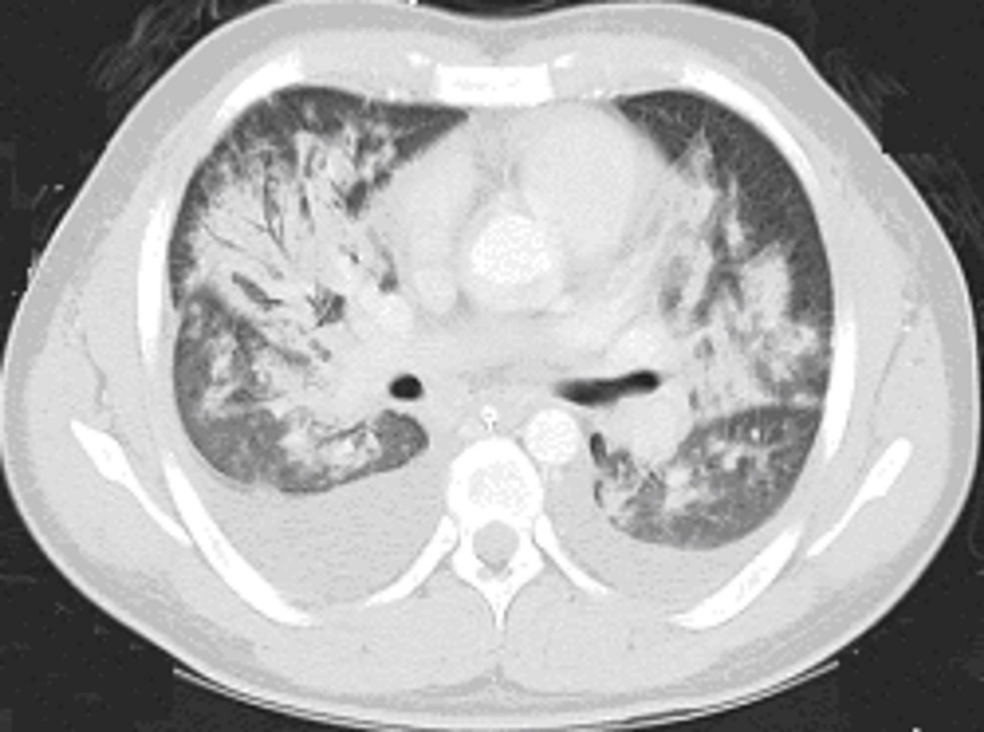
CT chest with IV contrast Axial chest CT demonstrates multifocal pneumonia throughout both lungs and bilateral pleural effusions CT: computed tomography; IV: intravenous

Initial blood cultures grew *Fusobacterium* spp. on day two and the patient’s antibiotics were changed to piperacillin-tazobactam and levofloxacin. His condition continued to deteriorate, and left neck tenderness continued to worsen. A CT of the neck revealed a thrombus in the left IJV (Figure 7) and a CT of the chest revealed bilateral lung nodules consistent with septic pulmonary emboli (Figure 8). On day three, the patient was transferred to the ICU and was emergently intubated to manage ensuing hypoxemic respiratory failure. He was then transferred to our facility for a higher acuity of care. Physical examination showed a swollen left neck. Pulmonary examination revealed bilateral rhonchi with decreased air-on-entry at the basis.

The antibiotic regimen was revised to incorporate piperacillin-tazobactam in addition to clindamycin. Heparin was also added to his regimen. He was extubated on hospital day seven and discharged on hospital Day 14 on oral amoxicillin-clavulanate and warfarin for three months. No data was obtained regarding clot progression and/or resolution.

Case 4

**Figure 9:**
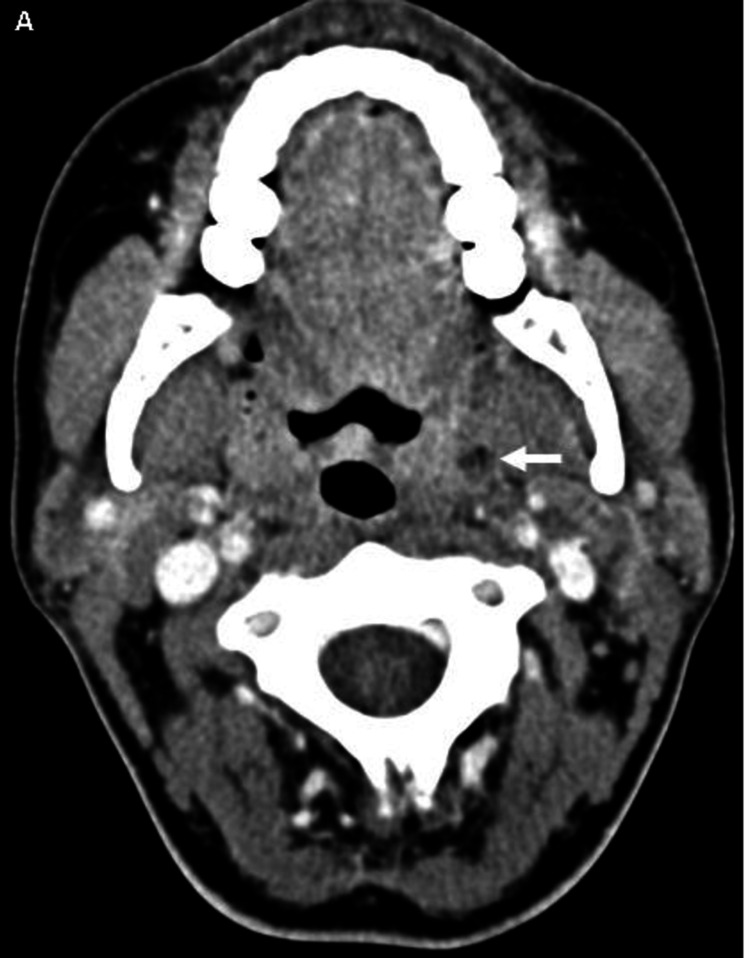
CT axial with IV contrast On axial contrast-enhanced CT, the left palatine tonsil demonstrates a small focus of low attenuation with an internal gas locule compatible with a small peritonsillar abscess (white arrowhead) CT: computed tomography; IV: intravenous

**Figure 10:**
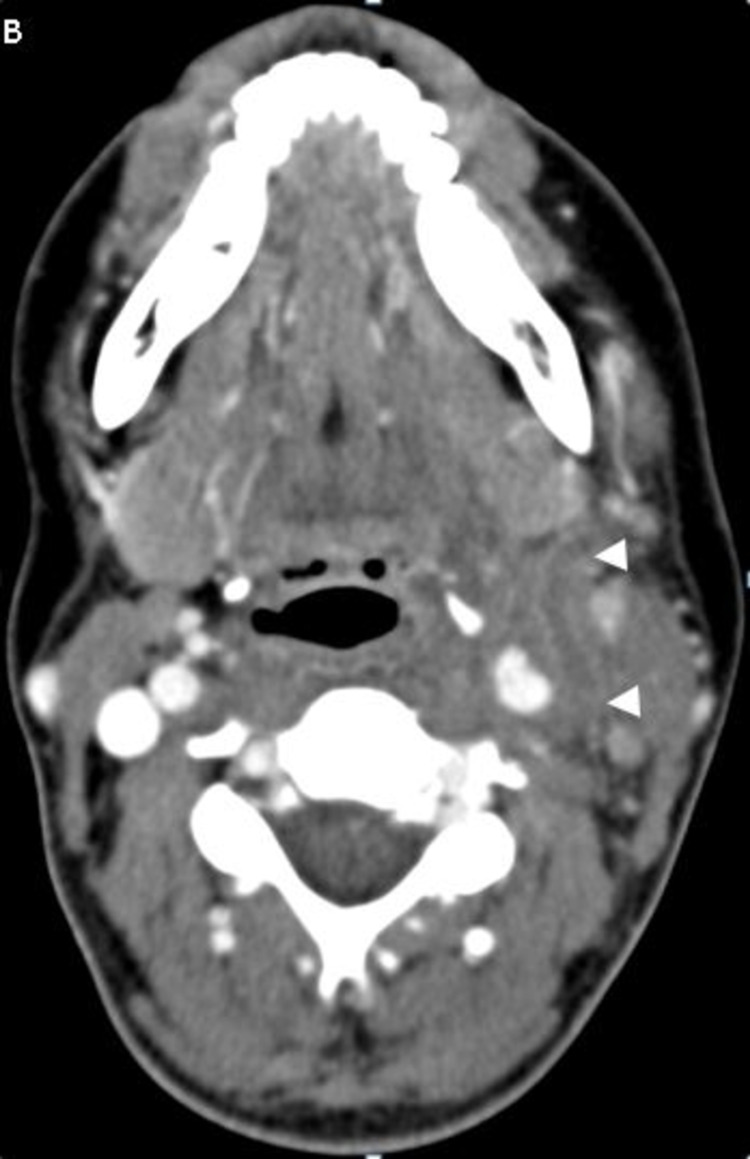
CT venogram of the neck CT image shows occlusion of the left internal jugular vein and left facial vein with surrounding fat stranding corresponding to areas of thrombophlebitis (white arrowheads) CT: computed tomography

A 30-year-old female with a past medical history notable for attention deficit and hyperactivity disorder, psoriasis, and depression initially presented to an outpatient urgent care center with a five-day history of left ear pain and odynophagia. She had a negative streptococcal RADT and was prescribed azithromycin and a five-day course of prednisone. Four days later, the patient was evaluated in the emergency department in a febrile state with progressive ear pain, muffled voice, swollen lymph nodes, and a temperature max of 103 °F. CT neck with IV contrast (Figure 9) demonstrated thrombosis of the left facial vein with enlargement of two cervical lymph nodes. Blood cultures grew group C streptococcus and *F. necrophorum*. The patient was initially prescribed cefepime and metronidazole and was de-escalated to ampicillin-sulbactam. On day two of hospitalization, a follow-up CT venogram of the neck (Figure 10) showed progression of thrombosis into the left IJV and left superior mediastinitis, with 6-mm fluid collection adjacent to the left palatine tonsil. A transthoracic echocardiogram demonstrated a 2-mm fibrinous echodensity on the right coronary cusp, which was classified as a fibrin strand. Apixaban was initiated as an anticoagulant on day five of hospitalization. The patient was medically optimized and discharged on day five with a 10-day course of IV ampicillin-sulbactam, followed by four more weeks of oral amoxicillin-clavulanate. Apixaban was continued for a total duration of three months. A follow-up CT venogram of the neck demonstrated persistent thrombosis of the left retromandibular vein and mid-segment of the IJV, as well as a lengthened occluded segment of the jugular vein without propagation of the clot. The patient remains on apixaban monotherapy pending further evaluation.

## Discussion

LS is a rare thrombotic condition resulting from an infection within the oropharyngeal space that manifests as septic thrombophlebitis of the IJV with the potential for metastatic septic emboli [1-16]. Many pathogens have been associated with LS; however, the most frequently isolated pathogens are *F. necrophorum* followed by other *Fusobacterium* spp. [4]. A prospective study from 1998 to 2001 estimated the annual incidence of LS to be 3.6 cases per one million persons [3,5]. However, the incidence was noted to be higher among patients aged 15-24 years (14.4 cases per one million per year) than among older patients, as was seen in our case series [3]. A follow-up study from 2010 to 2014 using a microbiology database highlighted similar findings within the age group of 15-24 years, reporting 9.4 cases per million per year [7].

There is a paucity of long-term population-based studies on LS, making it challenging to track the condition’s true incidence. While there are no data to suggest a true rise, there has been an increase in case reports and series related to *F. necrophorum* in the literature [2,4]. It is possible that this trend is due to better detection techniques in anaerobic blood cultures [4,9]. Other studies have suggested the pathogens are macrolide-resistant (specifically, erythromycin-resistant) [4,9]. Some patients were found to have been prescribed macrolide around the time of diagnosis [4,9]. It is also conceivable that simple tonsillitis could progress to LS in cases of true fusobacterial infection [2]. For this reason, some have linked the potential increase in LS to the declining rate of tonsillectomies [4].

The increasing cases of LS could also be due to the restriction of antibiotic use for sore throat [2,4,9]. There has been a general reluctance to prescribe antibiotics for the treatment of sore throat, with the streptococcal group A beta-hemolytic RADT often used to direct their use [10]. The RADT sensitivity ranges from 70-90% and the specificity from 90-100% [10]. Sore throat is a common presenting symptom encountered by primary care physicians, accounting for approximately 1-10% of office visits [11]. This clinical presentation should be viewed within the context of bacterial pharyngitis given that group A beta-hemolytic streptococcus (GABHS) is responsible for 5-15% of all cases of sore throat in adults [12].

LS generally affects healthy individuals after exposure to *F. necrophorum*, development of tonsillitis, and complication by peritonsillar abscess [1,2,4]. Other disease processes have also been associated with LS, such as mastoiditis, otitis media, and even blunt trauma [4]. In most cases, the presenting symptom of LS is a sore throat, which could erroneously lead to an incorrect diagnosis such as tonsillitis [4]. However, tonsillitis manifestations often completely resolve and are usually not associated with the signs of LS, such as generalized lymphadenopathy, thrombophlebitis, peritonsillar abscess, septicemia, deep neck infection, or cavitary lesions [4,15]. Patients with LS might also test positive for mononucleosis. In the earlier stages of LS, patients may present with oropharyngeal infection associated with febrile illness, rigors, and mononucleosis-like symptoms as seen in our case 3 [4,8]. The key distinguishing clinical features of LS are generalized cervical lymphadenopathy, as opposed to the unilateral cervical lymphadenopathy seen in mononucleosis [4]. Further, laboratory markers like elevated c-reactive protein (CRP) can rule out mononucleosis even if a monospot test is positive [4]. It is also conceivable that a patient might have concomitant LS and mononucleosis, even after a false-positive monospot test result [4].

It is critical to note that all of the patients in this series had a negative RADT. Worsening clinical symptoms should serve as a diagnostic consideration/indicator for LS [4,8]. Once the disease has progressed and metastasized, patients may experience further embolization and abscess formation in various organ systems, ultimately leading to organ damage [4,5,8].

There are several existing theories as to how LS occurs. Some groups have posited hematogenous spread via the tonsillar vein vs. lymphangitis, while others theorize that lymphadenitis as a primary focus extends into veins causing periphlebitis and endophlebitis with an associated thrombosis [4]. Still others hypothesize that progression occurs via an extension of the peritonsillar abscess into the deep connective tissue causing purulent periphlebitis and endophlebitis [4]. It has been shown in-vitro that *F. necrophorum* aggregates platelets during venous stasis resulting in coagulation [8]. Some researchers have also noted synergy between *F. necrophorum* and other pathogens such as Epstein-Barr virus, cytomegalovirus, and *Mycoplasma pneumoniae*, which may contribute to the disease progression [4]. Though the true mechanism remains unknown, infectious mononucleosis has been implicated in the development and progression of LS [4]. There is also a temporal association between the development of LS and the use of corticosteroids, which are often prescribed in the outpatient setting [4]. Cases 1 and 4 were prescribed a course of corticosteroids prior to the fulminant disease.

Prolonged antimicrobial therapy is the hallmark of LS treatment, using penicillin-based pharmacotherapy with metronidazole, as opposed to monotherapy with penicillin (given the possibility of resistance and polymicrobial infection) [2,4,15]. Metronidazole is highly favored over clindamycin given its stronger bactericidal activity and tissue penetration [4,15]. Antimicrobials should not be delayed if there is a strong clinical suspicion of LS, as early initiation of therapy has a direct effect on clinical outcomes [4]. The median duration of therapy is 42 days [4,15]. Only case 3 in this series had a prolonged course of anti-microbial therapy; the remaining cases were treated for an average duration of six weeks.

The use of anticoagulation for LS remains controversial. Currently, there are no randomized prospective controlled trials to assess the safety, efficacy, and associated mortality benefits (4,14) of anticoagulants in this setting. A temporal association has been reported with the use of anticoagulants and a faster dissolution of thrombus and shortened course of LS [4]. However, these studies were retrospective, observational, had a small patient population, or offered a final assessment with no direct mortality comparison [4,14,16]. A recent meta-analysis was attempted and did not demonstrate a statistically significant mortality benefit for patients with LS treated with anticoagulation vs. no treatment [16]. However, it is important to note that anticoagulation is used more liberally based on the location and extent of the clot (i.e., cavernous sinus thrombosis) [4]. In our series, only case 4 had a clot that was not relegated to the IJV (facial vein).

Currently, there are no specific guidelines regarding the use of anticoagulation for LS [4,14,16]. Surgical intervention such as IJV ligation and/or excision is recommended with the evidence of septic thrombophlebitis once all pharmacological therapies have been exhausted [4,6,8]. However, studies have not yet demonstrated a therapeutic success and/or mortality benefit [4]. Case 4 currently remains on anticoagulation and recent imaging has noted stable clot formation without progression and/or resolution. Surgical intervention has not been recommended.

As shown in this illustrative case series, the species most commonly identified in LS belong to the genus *Fusobacterium*, which does not grow well on the same aerobic medium used for other throat cultures. Each of the cases presented involved previously healthy patients, and all four had a negative streptococcal RADT. These patients were all admitted to the ICU due to septic shock and ARDS. The duration of antimicrobial therapy ranged from four to six weeks, with only two patients requiring anticoagulation. No surgical intervention was recommended for any of our patients despite medical admission to the ICU. One patient has had a true follow-up with recent imaging noting stable clot formation without resolution and/or progression (see Table 1 for the summary).

**Table 1:** Summary of cases RADT: rapid antigen detection test

Case number	RADT status	Monospot test	Duration of antibiotics (weeks)	Received anticoagulation	Surgical intervention	Clot location	Clot status at follow-up
1	Negative	Negative	6	No	No	Internal jugular vein	Unknown
2	Negative	Negative	6	No	No	Internal jugular vein	Unknown
3	Negative	Negative	15	Yes	No	Internal jugular vein	Unknown
4	Negative	Negative	6	Yes	No	Internal jugular vein and facial vein	Stable; no resolution

## Conclusions

LS is a thrombogenic disease that may become life-threatening if not recognized and treated early. The disease is often overlooked, leading to a delayed diagnosis in the setting of negative RADT and presumed mononucleosis. All of the patients in our series initially presented to an outpatient clinic. While most cases of pharyngitis are viral in etiology, primary care physicians must be mindful that a negative streptococcal RADT does not exclude a serious bacterial oropharyngeal infection. Prompt diagnosis and antibiotic treatment can halt the subsequent morbidity and mortality associated with this syndrome.
